# Hyperuricemia and deterioration of renal function in autosomal dominant polycystic kidney disease

**DOI:** 10.1186/1471-2369-15-63

**Published:** 2014-04-16

**Authors:** Miyeun Han, Hayne Cho Park, Hyunsuk Kim, Hyung Ah Jo, Hyuk Huh, Joon Young Jang, Ah-Young Kang, Seung Hyup Kim, Hae Il Cheong, Duk-Hee Kang, Jaeseok Yang, Kook-Hwan Oh, Young-Hwan Hwang, Curie Ahn

**Affiliations:** 1 Department of Internal Medicine, Seoul National University Hospital, Seoul, Korea; 2 Research Coordination Center for Rare Diseases, Seoul National University Hospital, Seoul, Korea; 3 Transplantation Research Institute, Seoul National University Medical Research Center, Seoul, Korea; 4 Department of Medicine, Program of Immunology, The Graduate School, Seoul National University, Seoul, Korea; 5 Department of Radiology, Seoul National University College of Medicine, Seoul, Korea; 6 Kidney Research Institute, Medical Research Center, Seoul National University College of Medicine, Seoul, Korea; 7 Department of Pediatrics and Adolescent Medicine, Seoul National University College of Medicine, Seoul, Korea; 8 Department of Internal Medicine, Ewha Womans University School of Medicine, Ewha Medical Research Center, Seoul, Korea; 9 Transplantation Center, Seoul National University Hospital, Seoul, Korea; 10 Department of Internal Medicine, Eulji General Hospital, Eulji University, 14 Hangeulbiseong-ro, Nowon-gu, Seoul 139-872, Korea; 11 Department of Internal Medicine, Seoul National University College of Medicine, 101 Daehak-Ro Jongno-Gu, Seoul 110-744, Korea

**Keywords:** Glomerular filtration rate, Hyperuricemia, Polycystic kidney, Autosomal dominant, Uric acid

## Abstract

**Background:**

The role of hyperuricemia in disease progression of autosomal dominant polycystic kidney disease (ADPKD) has not been defined well. We investigated the association of serum uric acid (sUA) with renal function and the effect of hypouricemic treatment on the rate of renal function decline.

**Methods:**

This is a single-center, retrospective, observational cohort study. A total of 365 patients with ADPKD who had estimated glomerular filtration rate (eGFR) ≥ 15 mL/min/1.73 m^2^ and who were followed up for > 1 year were included in our analysis. Hyperuricemia was defined by a sUA level of ≥ 7.0 mg/dL in male and ≥ 6.0 mg/dL in female or when hypouricemic medications were prescribed.

**Results:**

Hyperuricemia was associated with reduced initial eGFR, independent of age, sex, hypertension, albuminuria, and total kidney volume. During a median follow-up period of over 6 years, patients with hyperuricemia showed a faster annual decline in eGFR (−6.3% per year vs. −0.9% per year, p = 0.008). However, after adjusting for age, sex, hypertension and initial eGFR, sUA was no longer associated with either annual eGFR decline or the development of ESRD. Among 53 patients who received hypouricemic treatment, the annual eGFR decline appeared to be attenuated after hypouricemic treatment (pretreatment vs. posttreatment: −5.3 ± 8. 2 vs. 0.2 ± 6.2 mL/min/1.73 m^2^ per year, p = 0.001 by Wilcoxon signed-rank test).

**Conclusions:**

Although hyperuricemia was associated with reduced eGFR, it was not an independent factor for renal progression in ADPKD. However, the correction of hyperuricemia may attenuate renal function decline in some patients with mild renal insufficiency.

## Background

Uric acid has been regarded as a marker rather than a risk factor for the development of chronic kidney disease (CKD) because a low glomerular filtration rate (GFR) induces elevation of serum uric acid (sUA) level despite compensatory increases in urinary and gastrointestinal urate excretions. However, recent studies suggested an independent role of uric acid in the development of CKD. For instance, hyperuricemia is shown to be associated with an increased risk of CKD in large cohort studies such as the Atherosclerosis Risk in Communities and the Cardiovascular Health Study [1] and the Vienna Health Screening Project [2]. Moreover, hyperuricemia has been reported to be associated with the development of end-stage renal disease (ESRD) [3,4]. However, the pathogenic role of hyperuricemia in the progression of CKD is still controversial. Hyperuricemia has been reported as a risk factor for renal progression in IgA nephropathy [5], whereas sUA level was not associated with disease progression or kidney failure in general CKD population [6,7].

Association between autosomal dominant polycystic kidney disease (ADPKD) and hyperuricemia was first described by Rivera et al. [8]. ADPKD is frequently associated with hyperuricemia and gout [9], although fractional excretion of uric acid was not different from CKD groups of different etiologies [10,11]. Recent retrospective studies reported the association of high sUA levels with early-onset hypertension, large kidney volume, and increased risk of ESRD [12] or progression of renal dysfunction [13]. Considering that hyperuricemia can be a possible correctable risk factor for ADPKD progression, we postulated that 1) hyperuricemia is associated with concurrent renal function, 2) hyperuricemia contributes independently to deterioration of renal function, 3) and the correction of hyperuricemia attenuates renal function decline in ADPKD.

## Subjects and methods

### Study subjects

A total of 612 patients were screened at the ADPKD clinic in Seoul National University Hospital. ADPKD was diagnosed according to the unified criteria proposed by Pei et al. [14]. We selected 365 patients aged > 18 years with an estimated GFR (eGFR) of > 15 mL/min/1.73 m^2^ at the initial evaluation and who were followed up for > 1 year. Patients with certain conditions that can independently influence renal function such as diabetes [15], pregnancy, or malignancy were excluded from the analysis. The data were collected retrospectively from each patient between August 1999 and March 2012. The patients underwent a standardized evaluation including detailed family history, renal function, and computed tomography (CT) scan, which was obtained using a multidetector CT scanner (Somatom Sensation 16, Siemens; LightSpeed Ultra 8, GE; Brilliance CT 64, Philips; Somatom Definition, Siemens). The following clinical data and information were collected every 3–6 months: baseline epidemiologic profiles (age, sex, and body weight), medical history including diabetes, hypertension, and gout, medication history including antihypertensive medications and hypouricemic agents, blood pressure, and laboratory results (serum creatinine (sCr), sUA, hemoglobin, serum albumin levels, and urine dipstick). The CT scan was performed every 2 years and total kidney volume (TKV) was calculated using the modified ellipsoid method [16]. This study was approved by the Institutional Review Board of Seoul National University Hospital (H-1002-028-309). Informed consent was obtained from the subjects in accordance with the Declaration of Helsinki.

### Evaluation of renal function and hypertension

The sCr was measured at 3- or 6-month interval using the Jaffe method by Hitachi 7600 and Toshiba-200FR, which was calculated to Isotope Dilution Mass Spectrometry (IDMS)-traceable sCr. The Chronic Kidney Disease Epidemiology collaboration (CKD-EPI) formula was used to calculate eGFR. Delta eGFR (ΔeGFR/year) was calculated using the equation (recent eGFR − eGFR at initial visit)/follow-up duration (years). The CKD stage was classified according to the Kidney Disease Improving Global Outcomes (KDIGO) guidelines [17]. ESRD was defined by an eGFR of <15 mL/min/1.73 m^2^ or initiation of renal replacement therapy. Albuminuria was defined as a stick albumin level of >1+ by urine dipstick test. Urine albumin was quantified by the immunoturbidimetric assay using Toshiba-120FR. Hypertension was defined by a systolic blood pressure of >140 mmHg, diastolic blood pressure of >90 mmHg, or current use of antihypertensive medication.

### Uric acid measurement and hyperuricemia management

The sUA level was determined using the uricase method (Hitachi 7600 and Toshiba-200FR). Hyperuricemia was defined by a sUA level of ≥ 7.0 mg/dL in males and ≥ 6.0 mg/dL in females or when patients have received hypouricemic treatment. Hypouricemic medications were prescribed in patients with gout, a history of uric acid stones, or persistent elevation of sUA level of > 8.0 mg/dL that was not controlled by dietary modification in 2 successive visits.

### Statistical analyses

Continuous variables were expressed as mean ± standard deviation. Student *t* -test was used to compare the continuous variables between the groups. Linear regression analysis was used to find the association between sUA and clinical variables. Cox proportional hazard model was used to compare renal survival between groups. Wilcoxon signed-rank test was used to evaluate the effect of hypouricemic medication on the rate of renal progression before and after the start of medication. P < 0.05 was considered statistically significant. All the statistical analyses were performed using SPSS version 19.0 (SPSS Inc., Chicago, IL).

## Results

### Baseline clinical characteristics according to the presence of hyperuricemia

The clinical characteristics of 365 patients on initial evaluation are summarized in Table 1. They were divided into normouricemic (Group A, n = 278) and hyperuricemic (Group B, n = 87) groups. In Group B, 12 patients (3.3%) underwent hypouricemic treatment. The prevalence of hyperuricemia increased according to CKD stage as follows: 6.3% (6/95) in stage 1, 15.2% (28/184) in stage 2, 52.4% (33/63) in stage 3, and 87.0% (20/23) in stage 4.

**Table 1:** Baseline characteristics of participants at initial evaluation

	**Total (n = 365)**	Group A^* ^**(n = 278)**	**Group B**^**† **^**(n = 87)**	**p-value**
Male (%)	183 (50.1%)	122 (43.9%)	61 (70.1%)	< 0.001
Age (yrs)	43.5 ± 11.9	42.4 ± 11.3	47.1 ± 13.2	0.003
18-39	159 (43.6%)	128 (46.0%)	31 (35.6%)	
40-59	177 (48.5%)	133 (47.8%)	44 (50.6%)	
≥ 60	29 (7.9%)	17 (6.1%)	12 (13.8%)	
Follow up time (months)	73.5 ± 43.4	76.2 ± 43.5	64.7 ± 42.1	0.031
Hypertension (%)	257 (70.4%)	186 (66.9%)	71 (81.6%)	0.004
Systolic BP (mmHg)	136.4 ± 19.9	136.4 ± 20.3	136.3 ± 18.8	0.967
Diastolic BP (mmHg)	85.6 ± 13.9	86.1 ± 14.2	83.6 ± 12.8	0.190
Urinary stone (%)	96 (26.3%)	71 (25.5%)	25 (28.7%)	0.556
sUA (mg/dL)	5.51 ± 1.71	4.80 ± 1.11	7.76 ± 1.26	< 0.001
sCr (mg/dL)	1.17 ± 0.48	1.01 ± 0.23	1.67 ± 0.69	< 0.001
eGFR (ml/min/1.73 m^2^)	75.1 ± 24.1	81.6 ± 19.3	54.5 ± 26.3	< 0.001
CKD stage				< 0.001
Stage 1	95 (26.0%)	53 (19.1%)	6 (6.9%)	
Stage 2	184 (50.4%)	189 (68.0%)	28 (32.2%)	
Stage 3	63 (17.3%)	35 (12.6%)	33 (37.9%)	
Stage 4	23 (6.3%)	1 (0.4%)	20 (23.0%)	
Urine pH	6.03 ± 0.73	6.13 ± 0.73	5.74 ± 0.66	< 0.001
Dipstick albumin				0.017
None to 1+	342 (93.7%)	266 (95.7%)	76 (87.4%)	
> 1+	23 (6.3%)	12 (4.3%)	11 (12.6%)	
TKV (mL)	1,524 ± 1,171	1,416 ± 1,050	1,963 ± 1,500	0.013
Losartan (%)	58 (15.9%)	50 (18.0%)	8 (9.2%)	0.025
Diuretic (%)	36 (9.9%)	27 (9.7%)	9 (10.3%)	0.863

^*^Group A : sUA < 7.0 mg/dL (Male) or sUA < 6.0 mg/dL (Female), ^†^Group B: sUA ≥ 7.0 mg/dL (Male) or sUA ≥ 6.0 mg/dL (Female) or on hypouricemic medication. BP, blood pressure; sUA, serum uric acid; sCr, serum creatinine; eGFR, estimated glomerular filtration rate; CKD, chronic kidney disease; TKV, total kidney volume.

When compared with those in Group A, the patients in Group B were slightly older (47.1 ± 13.2 vs. 42.4 ± 11.3 years, p = 0.003), had higher prevalence of hypertension (81.6% vs. 66.9%, p = 0.004), showed male predominance (70.1% vs. 43.5%, p < 0.001), and had shorter follow-up duration (64.7 ± 42.1 vs. 76.2 ± 43.5 months, p = 0.031). Group B had higher sCr levels (1.67 ± 0.69 vs. 1.01 ± 0.23 mg/dL, p < 0.001) and lower eGFR (54.5 ± 26.3 vs. 81.6 ± 19.3 mL/min/1.73 m^2^, p < 0.001) compared to Group A.

### Hyperuricemia was negatively correlated with initial eGFR

**Figure 1:**
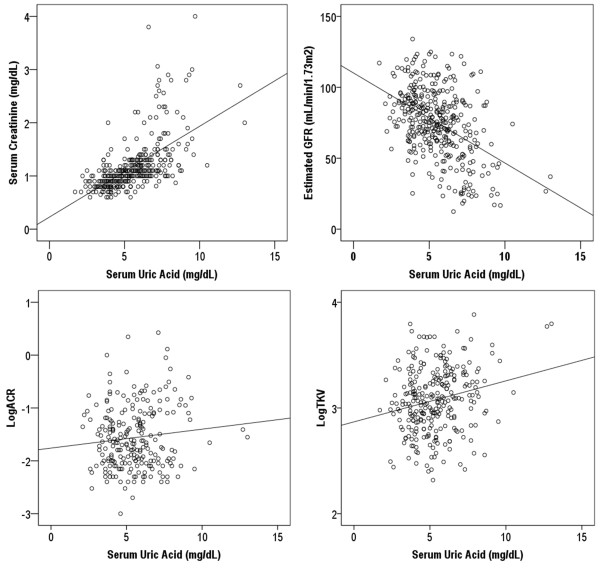
**The association of serum uric acid level with serum creatinine, eGFR, albuminuria and TKV.** Relationship between serum uric acid (sUA) level with serum creatinine (sCr) (*R*^2^ = 0.370, p < 0.001), eGFR (*R*^2^ = 0.202, p < 0.001), Log albumin-to-creatinine ratio (ACR) (*R*^2^ = 0.011, p = 0.111) and LogTKV (*R*^2^ = 0.045, p < 0.001) in ADPKD patients. The sCr and eGFR was measured in 365 patients, ACR in 230 patients and TKV in 278 patients. eGFR, estimated glomerular filtration rate; TKV, total kidney volume; ADPKD, autosomal dominant polycystic kidney disease.

Associations between sUA and sCr levels, eGFR, TKV, and albumin to creatinine ratio (ACR) were analysed (Figure 1). The sUA level was positively correlated with sCr level (*R*^2^ = 0.370, p < 0.001), ACR (*R*^2^ = 0.011, p = 0.111) and TKV (*R*^2^ = 0.045, p < 0.001) but negatively correlated with eGFR (*R*^2^ = 0.202, p < 0.001). Simple linear regression analysis in 353 patients without hypouricemic treatments at the initial evaluation revealed that age, albuminuria, TKV, and sUA level were correlated with reduced initial eGFR. After adjustment for age, male sex, blood pressure, albuminuria, and TKV, sUA levels still remained a significant factor (β = −5.117, p < 0.001; Table 2).

**Table 2:** Factors associated with eGFR in ADPKD patients^*^

	** Univariate**		** Multivariate**	
	**Beta ± SE**	**p-value**	**Beta ± SE**	**p-value**
Age (yr)	−1.208 ± 0.087	< 0.001	−0.986 ± 0.086	< 0.001
Female (vs. male)	−2.714 ± 2.520	0.282	−9.217 ± 2.224	< 0.001
Mean BP (mm Hg)	−0.229 ± 0.089	0.010	0.043 ± 0.073	0.558
sUA (mg/dL)	−6.282 ± 0.666	< 0.001	−5.117 ± 0.666	< 0.001
Albuminuria	−14.687 ± 5.160	0.005	−4.956 ± 3.890	0.204
TKV (mL)^†^	−19.524 ± 4.060	< 0.001	−12.782 ± 3.245	< 0.001

^*^353 patients without hypouricemic medication. ^†^transformed in logarithmic scale. eGFR, estimated glomerular filtration rate; ADPKD, autosomal dominant polycystic kidney disease; SE, standard error; BP, blood pressure; sUA, serum uric acid; TKV, total kidney volume.

### Hyperuricemia was not independently associated with annual eGFR decline and ESRD progression

Among 365 patients, 42 patients (11.5%; 20 in Group A and 22 in Group B) progressed to ESRD during the follow-up (mean duration, 73.5 ± 43.4 months). When evaluating the deterioration of eGFR, we examined 296 patients not taking hypouricemic medication (255 patients from Group A and 41 patients from Group B) to exclude the influence of hypouricemic agents (Table 3). The mean sUA level was 4.69 ± 1.08 mg/dL in normouricemic Group A and 7.61 ± 0.95 mg/dL in Group B (p < 0.001), indicating a difference of 2.9 mg/dL. Progression to ESRD occurred in 14 (34.1%) out of 41 patients in Group B and 14 (5.4%) out of 255 patients in Group A (p < 0.001). Group B showed a faster annual decline in eGFR (ΔeGFR/year) than Group A in absolute (−1.87 ± 3.30 vs. −0.29 ± 4.22 mL/min/1.73 m^2^ per year, p = 0.026) and relative values (−6.23% ± 9.84% vs. −0.72% ± 6.03% per year, p = 0.001).

**Table 3:** Annual eGFR decline (ΔeGFR) in ADPKD patients according to the presence of hyperuricemia^*^

	**Group A**^**†**^**(n = 255)**	**Group B**^**‡**^**(n = 41)**	**p-value**
Mean sUA level (mg/dL)	4.69 ± 1.08	7.61 ± 0.95	< 0.001
F/U time (months)	73.0 ± 42.2	62.0 ± 46.8	0.134
Initial eGFR (mL/min/1.73 m^2^)	82.0 ± 19.6	52.2 ± 27.4	< 0.001
Final eGFR (mL/min/1.73 m^2^)	75.6 ± 27.9	48.3 ± 31.7	< 0.001
ΔeGFR (mL/min/1.73 m^2^/yr)	−0.29 ± 4.22	−1.87 ± 3.30	0.026
ΔeGFR (%/yr)	−0.72 ± 6.03	−6.23 ± 9.84	0.001
ESRD progression, n (%)	14 (5.4%)	14 (34.1%)	< 0.001

^*^A total of 296 patients without hypouricemic medications were included in the analysis. ^†^Group A: sUA < 7.0 mg/dL (Male) or < 6.0 mg/dL (Female), ^‡^Group B: sUA ≥ 7.0 mg/dL (Male) or ≥ 6.0 mg/dL. sUA, serum uric acid; ADPKD, autosomal dominant polycystic kidney disease; F/U, follow-up; eGFR, estimated glomerular filtration rate; ESRD, end-stage renal disease.

Linear regression analysis was performed to determine the factors influencing ΔeGFR/year. In univariate analysis, age, hypertension, initial eGFR, and sUA level were significantly associated with ΔeGFR/year. However, after adjusting for age, sex, blood pressure, and initial eGFR, neither sUA level nor hyperuricemia was significantly associated with ΔeGFR/year (Table 4). In the same manner, multivariate Cox regression analysis showed that hyperuricemia was not an independent risk factor for ESRD (Table 5).

**Table 4:** Factors affecting annual eGFR decline (ΔeGFR) in patients with ADPKD

	** Univariate**		** Multivariate**	
	**Beta ± SE**	**p-value**	**Beta ± SE**	**p-value**
Age (yr)	−0.067 ± 0.020	0.001	−0.071 ± 0.034	0.036
Female	0.369 ± 0.491	0.453	0.775 ± 0.731	0.290
Mean BP (mm Hg)	−0.065 ± 0.018	0.001	−0.056 ± 0.019	0.003
History of stone	0.260 ± 0.377	0.491	-	-
sUA (mg/dL)	−0.330 ± 0.165	0.047	−0.212 ± 0.275	0.443
Initial eGFR (mL/min/1.73 m^2^)	0.036 ± 0.010	0.001	−0.001 ± 0.020	0.971

eGFR, estimated glomerular filtration rate; ADPKD, autosomal dominant polycystic kidney disease; BP, blood pressure; sUA, serum uric acid; SE, standard error.

**Table 5:** Multivariate cox regression for development of end-stage renal disease

	**Hazard ratio**	** 95% CI**	**p-value**
Age (year)	0.931	0.864, 1.003	0.060
Female	3.664	0.011, 1239.509	0.662
Initial eGFR (mL/min/1.73 m^2^)	0.720	0.620, 0.836	< 0.001
Mean BP (mm Hg)	1.023	0.978, 1.070	0.331
Hyperuricemia (Group B vs. A)	3.082	0.008, 1259.413	0.714

Group A: sUA <7.0 mg/dL, Group B: sUA ≥7.0 mg/dL. CI, confidence interval; eGFR, estimated glomerular filtration rate; BP, blood pressure.

### Correction of hyperuricemia may attenuate renal function decline

Although we were not able to show the independent association of sUA level with renal progression in ADPKD patients who were not taking hypouricemic agents, we did exploratory analysis in a subgroup of our cohort to investigate the effect of hypouricemic treatment on eGFR. Among 57 patients newly starting hypouricemic treatment during follow-up period, 53 patients had follow-up period more than 1 year of pre- and post-treatment. Serum UA and eGFR were measured every 3 months. The slopes of the annual change in eGFR before and after hypouricemic treatment were analyzed in 53 patients receiving either allopurinol (n = 12) or benzbromarone (n = 41) more than a year.

**Figure 2:**
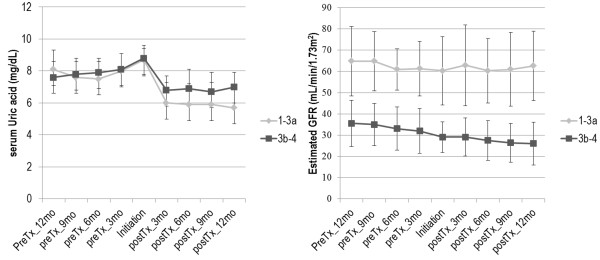
**Serial change in sUA and eGFR before and after hypouricemic treatment.** After initiation of hypouricemic treatment, mean sUA level decreased from 8.7 ± 0.9 to 5.7 ± 1.4 mg/dL in CKD stage 1-3a group, whereas from 8.8 ± 0.6 to 7.0 ± 0.9 mg/dL in CKD stage 3b-4 group. Mean eGFR level increased from 60.3 ± 16.1 to 62.6 ± 16.4 mL/min/1.73 m^2^ in CKD stage 1-3a group, whereas eGFR decreased from 29.1 ± 7.2 to 26.0 ± 10.0 mL/min/1.73 m^2^ in CKD stage 3b-4 group.

Among them, 13 patients were included in CKD stage 1 or 2, 19 in CKD stage 3a, 11 in CKD stage 3b, and 10 in CKD stage 4 before treatment initiation. Mean sUA was 8.70 ± 0.78 mg/dL and mean eGFR was 47.9 ± 20.3 mL/min/1.73 m^2^ at the time of hypouricemic treatment initiation. The change in sUA and eGFR before and after 1 year of treatment initiation was analyzed (Table 6). After the hypouricemic treatment, the annual decline of renal function (ΔGFR/year) slowed from −5.3 ± 8.2 to 0.2 ± 6.2 mL/min/1.73 m^2^ per year (Wilcoxon signed-rank test, p = 0.001). Further analysis showed that correction of hyperuricemia may attenuate renal function decline in early CKD stages (1 ~ 3a) whereas it may not attenuate renal function decline in advanced CKD stages (3b ~ 4). Serial changes in sUA and eGFR are presented in Figure 2 and Additional file 1: Table S1. There was no difference between allopurinol and benzbromarone group (data not shown). However, significant changes in systolic blood pressure were also observed after hypouricemic treatment (Additional file 1: Table S2). Because lowering blood pressure itself and change of anti-hypertensive medication (32.1% during the observation period), especially of renin-angiotensin system blockers may affect eGFR, the effect of blood pressure on eGFR was examined by using generalized estimating equation, showing that no significant effect over 2-year observation period (data not shown). In summary, blood pressure changes did not significantly influence eGFR changes after hypouricemic treatment.

**Table 6:** Annual eGFR change (ΔeGFR) before and after hypouricemic treatment

**CKD stage**	**PreTx (1 yr)**	**Initiation of Tx**	**PostTx (1 yr)**	p-value^*^
Total (n = 53)				
sUA (mg/dL)	7.88 ± 1.09	8.70 ± 0.78	6.22 ± 1.40	
eGFR (mL/min/1.73 m^2^)	53.2 ± 20.3	47.9 ± 20.3	48.1 ± 22.9	
ΔeGFR (mL/min/1.73 m^2^/yr)	−5.3 ± 8.2		0.2 ± 6.2	0.001
Stage 1-3a (n = 32)				
sUA (mg/dL)	8.06 ± 1.15	8.66 ± 0.90	5.70 ± 1.41	
eGFR (mL/min/1.73 m^2^)	64.8 ± 16.3	60.3 ± 16.1	62.6 ± 16.4	
ΔeGFR (mL/min/1.73 m^2^/yr)	−4.5 ± 9.4		2.3 ± 5.7	0.001
Stage 3b-4 (n = 21)				
sUA (mg/dL)	7.62 ± 0.97	8.77 ± 0.59	7.03 ± 0.94	
eGFR (mL/min/1.73 m^2^)	35.6 ± 10.9	29.1 ± 7.2	26.0 ± 10.0	
ΔeGFR (mL/min/1.73 m^2^/yr)	−6.6 ± 5.7		−3.1 ± 5.6	0.465

*Wilcoxon signed-rank test. eGFR, estimated glomerular filtration rate; CKD, chronic kidney disease; Tx, treatment; sUA, serum uric acid.

## Discussion

In our retrospective cohort study, we demonstrated that sUA level is associated with renal function in ADPKD. However, we were not able to show that hyperuricemia independently contributes to the renal progression.

In our study, sUA level was associated with concurrent eGFR, independent of age, sex, albuminuria, and TKV. This result is consistent with those from previous studies reporting that hyperuricemia is associated with early-onset hypertension, TKV, and increased risk of ESRD [12]. Hyperuricemia also showed negative correlation with ΔeGFR/year. However, after adjusting age, sex, blood pressure, and initial eGFR, the influence of hyperuricemia was not statistically significant. This result may be partly explained by the small difference in sUA level between patients with and without hyperuricemia because we excluded high-risk patients with hyperuricemia who were already being treated. Moreover, the follow-up duration was relatively short. The median follow-up duration was only 73.5 months, and 41.6% of the patients were followed up for <5 years.

Although we failed to show independent effect of hyperuricemia on renal progression, we examined the effect of hypouricemic medication on the slope of annual eGFR change. Correction of hyperuricemia appeared to attenuate ΔeGFR/year, especially for the patients in early CKD stages (1~3a), suggesting that hyperuricemia may influence renal function deterioration in some patients. Although our result is limited by small number of patients and short observation time, this is consistent with the previous finding that allopurinol therapy preserved sCr level and lowered the risk of renal progression in hyperuricemic patients with mild to moderate CKD [18,19]. On the contrary, hypouricemic treatment had no effect on preserving eGFR in patients with CKD stage 3b to 4. This lack of effect may be related to the lower efficacy of uricosuric agents (e.g. benzbromarone) in the advance kidney failure. Mean sUA level decreased from 8.9 ± 0.9 mg/dL to 5.7 ± 1.4 mg/dL in stage 1-3a group, whereas from 8.8 ± 0.6 mg/dL to 7.0 ± 0.9 mg/dL in stage 3b-4 group. The effect of hypouricemic agents for renal function preservation in advanced CKD stage should be evaluated with more potent hypouricemic agents such as febuxostat in long-term prospective studies.

Several mechanisms were proposed to explain renal dysfunction by hyperuricemia. First, association between increased sUA level and cardiovascular disease has been reported [20]. In our study, no difference in cardiovascular event was observed between the normouricemic and hyperuricemic groups (data not shown). However, the possibility of hyperuricemia causing renal function decline through cardiovascular events cannot be excluded because of the small sample size and short-term follow-up [21]. Second, hyperuricemia may induce direct renal injury through the activation of the renin-angiotensin aldosterone system (RAS). Renal cyst enlargement in ADPKD is known to be associated with stimulation of the circulating and intrarenal RAS [22]. Helal et al. [12] speculated that endothelial dysfunction, which is a well-known characteristic of ADPKD [23], and activation of RAS induced by hyperuricemia would contribute to the progression of ESRD in ADPKD. Endothelial dysfunction and early-onset hypertension are important prognostic factors for the deterioration of renal function in ADPKD [24]. In addition, soluble uric acid might activate inflammatory pathways such as tumor necrosis factor alpha (TNF-α) and chemokines [25] and C-reactive protein [26], possibly leading to interstitial fibrosis. In CKD patients, association between hyperuricemia and increased urinary transforming growth factor beta (TGF-β) was also reported [27].

The pathogenic role of hyperuricemia in renal progression needs to be further investigated in regard to urate handling in ADPKD. In ADPKD, altered tubular membrane transport process might affect renal urate handling and homeostasis. Compared to general population, a higher prevalence of uric acid stone was noted in ADPKD. However, previous studies showed inconsistent data about urate handling in ADPKD [10,11]. We also performed immunohistochemical staining of four major urate transporters (*URAT1, GLUT9, NPT4*, and *OAT3*) in 3 ADPKD kidneys and 1 normal control kidney, which revealed strong expression of all 4 urate transporters along the compressed proximal tubules nearby renal cysts [28] (Additional file 2: Figure S1). It may suggest the possibility of altered urate handling in ADPKD that may lead to hyperuricemia. However, our result needs to be validated through functional study in a large number of samples.

Our study has several limitations. This is a single-center, retrospective cohort study. Therefore, further prospective study is warranted to evaluate the causal relationship between hyperuricemia and renal function decline. In addition, genetic factor, which is the strongest predictor of renal progression, has not been evaluated in this study. In the analysis of the hyperuricemia and annual eGFR decline, mixed effects model or generalized estimating equation model would be best suited. However, they could not be used due to some missing data of sUA and eGFR during follow-up period and the interval of measurement was not regular. Lastly, we failed to show the independent effect of hyperuricemia on renal progression.

## Conclusions

In conclusion, we failed to show the independent effect of hyperuricemia on renal progression. Nevertheless, for the first time, we demonstrated the correction of hyperuricemia with uric acid lowering agents may attenuate renal progression in the early CKD stages suggesting that treatment of hyperuricemia would be beneficial for some ADPKD patients to preserve renal function. Further prospective study is needed to verify the impact of hyperuricemia control on disease progression in ADPKD.

## Abbreviations

ACR: Albumin to creatinine ratio; ADPKD: Autosomal dominant polycystic kidney disease; CKD: Chronic kidney disease; CKD-EPI: Chronic kidney disease epidemiology collaboration; CT: Computed tomography; ESRD: End-stage renal disease; GFR: Glomerular filtration rate; GWAS: Genome-wide association studies; IDMS: Isotope dilution Mass Spectrometry; KDIGO: Kidney disease improving global outcomes; RAS: Renin-angiotensin aldosterone system; sCr: Serum creatinine; sUA: Serum uric acid; TGF-β: Transforming growth factor beta; TKV: Total kidney volume; TNF-α: Tumor necrosis factor alpha.

## Competing interests

The authors declare that they have no competing interests.

## Authors’ contributions

MH collected the data and drafted the manuscript. HP participated in the design of the study and drafted the manuscript. HK participated in the acquisition of data. HC, HH participated in the analysis and interpretation of data. JJ, AK carried out immunohistochemical staining. SK carried out kidney volume measurement. HC, DK participated in critical revising the manuscript. JY, KO participated in the design of the study and interpretation of data. YH helped interpretation of data and drafted the manuscript. CA conceived of the study, interpretation of data and revising the manuscript. All authors read and approved the final manuscript.

## Pre-publication history

The pre-publication history for this paper can be accessed here:

http://www.biomedcentral.com/1471-2369/15/63/prepub

## Supplementary Material

Additional file 1: Table S1 Serum uric acid and eGFR pre-/post-treatment 1 year. **Table S2.** Average systolic, diastolic, and mean blood pressure at 1 year pre-and post-treatment. Click here for file

Additional file 2: Figure S1 Immunohistochemcalstaining of URAT1, GLUT9, NPT4 and OAT3. Click here for file
