# Supplementary material for: Hyperuricemia and deterioration of renal function in autosomal dominant polycystic kidney disease
Source: BMC Nephrol. 2014 Apr 16;15:63. doi: 10.1186/1471-2369-15-63 (PMC4021172; doi:10.1186/1471-2369-15-63)
Supplement: Additional file 1: Table S1 — Serum uric acid and eGFR pre-/post-treatment 1 year. Table S2. Average systolic, diastolic, and mean blood pressure at 1 year pre-and post-treatment. [file 1471-2369-15-63-S1.pdf]

Additional File

Supplementary table 1. Serum uric acid and eGFR pre-/post-treatment 1 year

	PreTx_12mo	preTx_9mo	preTx_6mo	preTx_3mo	Initiation	postTx_3mo	postTx_6mo	postTx_9mo	postTx_12mo
Total (n)	53	48	49	46	53	52	51	51	53
SUA	7.9 ± 1.1	7.7 ± 1.0	7.6 ± 1.0	8.1 ± 1.1	8.7 ± 0.8	6.3 ± 1.2	6.3 ± 1.3	6.2 ± 1.4	6.2 ± 1.4
eGFR	53.2 ± 20.3	52.7 ± 19.3	49.1 ± 17.1	47.9 ± 18.9	47.9 ±20.3	49.3 ± 22.9	46.8 ± 20.8	46.8 ± 22.4	48.1 ± 22.9
Stage 1-3a (n)	32	29	28	25	32	31	30	30	32
SUA	8.1 ± 1.2	7.6 ± 1.0	7.5 ± 1.1	8.0 ± 1.1	8.7 ± 0.9	6.0 ± 1.3	5.9 ± 1.3	5.9 ± 1.4	5.7 ± 1.4
eGFR	64.8 ± 16.3	64.8 ± 14.0	61.0 ± 9.7	61.3 ± 12.9	60.3 ± 16.1	62.9 ± 19.0	60.3 ± 15.1	61.0 ± 17.3	62.6 ± 16.4
Stage 3b-4 (n)	21	19	21	21	21	21	21	21	21
SUA	7.6 ± 1.0	7.8 ± 1.0	7.9 ± 0.89	8.1 ± 1.0	8.8 ± 0.6	6.8 ± 0.9	6.9 ± 1.2	6.7 ± 1.2	7.0 ± 0.9
eGFR	35.6 ± 10.9	35.0 ± 10.0	33.1 ± 10.2	32.0 ± 10.6	29.1 ± 7.2	29.1 ± 9.0	27.5 ± 9.3	26.4 ±9.2	26.0 ± 10.0

Tx, treatment; mo, month

Supplementary table 2. Average systolic, diastolic, and mean blood pressure at 1 year pre- and post-treatment

	Pre-treatment*	Post-treatment*	p-value (paired-t)
SBP	132.4 ± 10.6	128.8 ± 8.6	0.006
DBP	83.3 ± 7.9	80.1 ± 6.5	0.077
MBP	99.7 ± 8.4	96.3 ± 6.8	0.012

*The average blood pressure measured every 3 months during 1 year of pre- and post-treatment, respectively

Supplementary Figure 1. Immunohistochemcal staining of URAT1, GLUT9, NPT4 and OAT3

Expression of several urate transporters, reabsorption (URAT1, GLUT9) and excretion (NPT4, OAT3), are shown (x40 and x200 (inlet)). Uric acid transporters were strongly stained in proximal tubular epithelial cells in ADPKD tissue. All the transporters except URAT1 are also expressed in some of the cyst-lining epithelial cells. ADPKD; autosomal dominant polycystic kidney disease.
