# Supplementary figures and images for: Hyperuricemia and deterioration of renal function in autosomal dominant polycystic kidney disease
Source: BMC Nephrol. 2014 Apr 16;15:63. doi: 10.1186/1471-2369-15-63 (PMC4021172; doi:10.1186/1471-2369-15-63)

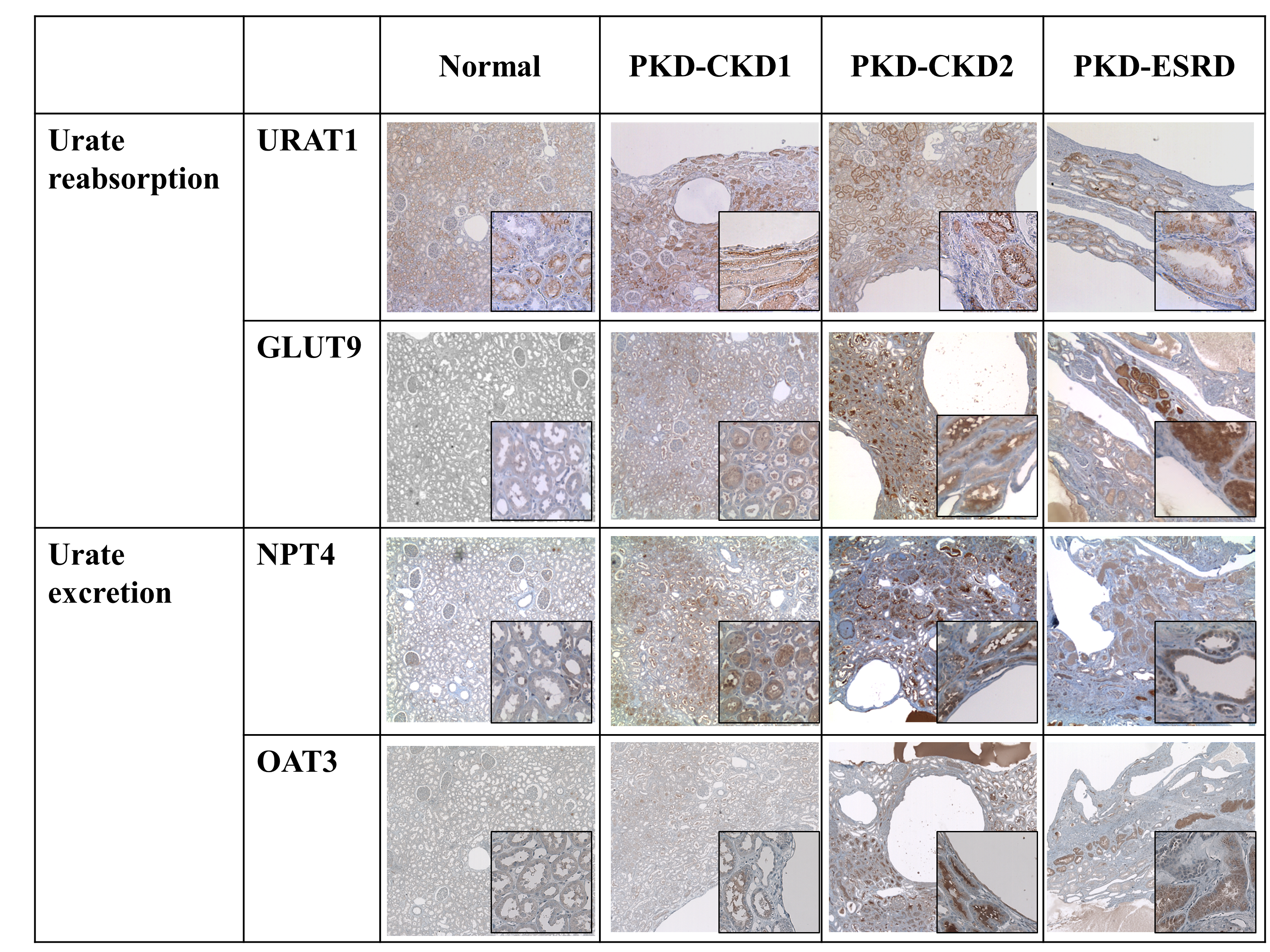

Supplement: Additional file 2: Figure S1 — Immunohistochemcalstaining of URAT1, GLUT9, NPT4 and OAT3. [file 1471-2369-15-63-S2.tiff]
